# Association of food intake with sleep disorders in children and adolescents with obesity

**DOI:** 10.1016/j.sleepx.2022.100053

**Published:** 2022-08-23

**Authors:** Raquel S.M. Zarpellon, Dra Regina M. Vilela, Fernando Mazzilli Louzada, Dra Rosana B. Radominski, Dra Ana Chrystina de Souza Crippa

**Affiliations:** a Postgraduate Program in Child and Adolescent Health, Hospital de Clínicas, Federal University of Paraná, Curitiba, Paraná, Brazil; b Federal University of Paraná (UFPR), Health Sciences Sector, Department of Nutrition, Curitiba, Paraná, Brazil; c Federal University of Paraná (UFPR), Department of Physiology, Curitiba, Paraná, Brazil; d Federal University of Paraná (UFPR), Health Sciences Sector, Curitiba, Paraná, Brazil; e Neuropediatrics Center (CENEP), Hospital de Clínicas, Federal University of Paraná, Curitiba, Paraná, Brazil

**Keywords:** Pediatric obesity, Sleep disturbances, Parasomnias, SDSC

## Abstract

**Introduction:**

the great increase in childhood obesity rates is well documented in the scientific literature. However, the influence of diet on sleep quality in children and adolescents still needs further research in order to be better understood. The objective of this study was to evaluate the association between diet and sleep characteristics and in children and adolescents with obesity.

**Methods:**

observational analytical cross-sectional study with prospective data collection. Forty-three children and adolescents aged between 6 and 13 years diagnosed with obesity and treated at a public tertiary care institution participated in the study. The 6-day Food Intake Registry was used to evaluate the intake of energy and macronutrients. To investigate the risk of sleep disturbances and to know the routine and characteristics of sleeping habits, the Sleep Disturbance Scale for Children (SDSC) questionnaire was used.

**Results:**

and discussion: Food intake showed association with Sleep Breathing Disorders (SBD) and Sleep Wake Transition Disorders (SWTD). The sum of all SDSC factors demonstrated the presence of pathological sleep in most patients (n = 25).

## Highlights

• Positive association between food intake and SBD through the analysis of the SDSC.
• Positive association between food intake and SWTD through the analysis of the SDSC.
• Positive association between food intake and the risk for sleep disorders.

## 1 Introduction

When studying the aspects inherent to sleep, it is observed that growth [1,2] and development [1] in childhood and adolescence may be affected by sleep disturbances. The worsening in the quality of sleep and the reduction in the sleep duration may be factors that have contributed to the increase in energy intake [[2], [3], [4]], childhood obesity [5,6], leading to consequences in several metabolic functions [7]. In the literature, it is well-founded the association of childhood obesity and energy intake with lower quantity of sleep, as well as a worsening in its quality, in relation to difficulty in initiating sleep [4,5], sleep breathing disorders [5,7], higher number of night awakenings [4], sleeping later [3,8] and waking up earlier [5].

Higher than recommended calorie intake and difficulty in maintaining healthy eating habits may be associated with lower amount and quality of sleep in children and adolescents [9]. At the same time, children and adolescents who sleep less and later may tend to consume more energy in the evening [7]. Regarding the intake of macronutrients, the lack of sleep may lead to a higher intake of lower nutritional quality foods, with higher amount of fat, more processed and with higher Glycemic Index [6]. Low protein intake (<16% of VET) may be associated with difficulty initiating sleep while high protein intake (>19% of VET) may lead to difficulty maintaining sleep [10]. The higher intake of sugary foods had a negative association with the quality of sleep [11] and the lower number of hours of sleep may be related to the higher intake of soft drinks and sweets in general [7]. Additionally, it was reported that low carbohydrate intake (<50% of VET) was associated with difficulty in maintaining sleep, at the same time the presence of sleep disorders such as insomnia, obstructive sleep apnea or the combination of the two, were related to lower carbohydrate intake when compared to those who did not have the disorder or with non-obese people [10].

The factors that may contribute to the increase in childhood obesity (e.g., increased consumption of fatty foods and foods rich in simple carbohydrates, sugary drinks, and sedentary lifestyle in children, among others) are well known. It is possible to add the worsening in the quality and quantity of sleep in the last two decades, for playing an important role regarding food choices, which may lead the patient with sleep disorders to develop obesity [1,2,4,9,12,13]. Behavioral studies reinforce that sleep deprivation can lead to increased binge eating [14], as well as the search for foods with a more pleasant taste, with higher amounts of sugar and fat [13]. Improving sleep quality may become an alternative to achieve more promising results in the prevention and reduction of pediatric obesity, since it could promote the choice for a healthier and balanced diet [2,4,8].

Another factor to be considered is the effect of physical activity on sleep, in which irregular sleep with shorter duration at night can affect the rhythm of physical activity the next day [15] and sleep duration in children was negatively associated with physical activity [6], as insufficient sleep can increase body fatigue, making it difficult to practice physical activity with an increase in sedentary lifestyle [9,15], thus creating a conditioning habit increasing the risk of obesity [16,17].

Considering that research focused on the impact of sleep disorders on food intake have been explored and considering the scarcity of studies assessing the impact of food on sleep quality in children and adolescents with obesity, this study aims to evaluate the association between energy and macronutrient intake with sleep characteristics.

## 2 Methods

The registration of patients as well as data collection occurred in the period from March to December 2019. The sample of this research is a convenience sample, in which, it was estimated that only seventy-five patients met the inclusion criteria of the study. Forty-nine patients accepted to participate in the study, but only forty-three completed the study according to the inclusion and exclusion criteria described above. The study protocol was approved by the Ethics Committee of the institution where the research was conducted under CAAE: 89706518.1.0000.0096 and all participants or their guardians signed the Consent and Informed Consent Form.

This is an analytical observational cross-sectional study with prospective data collection that involved children and adolescents aged 6–13 years of both sexes, treated at an outpatient clinic of the public health system. As inclusion criteria, patients should have the diagnosis of obesity by the criterion of the percentile bigger than P 97 [18] and evaluated according to the WHO growth guidelines [19], whose parents or guardians signed the Informed Consent Term. The exclusion criteria for this study were failure to complete the 6-day Food Consumption Record and the presence of mental or intellectual impairment in children and adolescents**.**

### 2.1 Data collection

The consultations were scheduled in advance and, according to the data of the patients in the institution's records that were in accordance with the inclusion criteria of this research, they were pre-selected after passing through the nursing service to check the anthropometric data according to the methods established by the proposed Ministry of Health. The interviews were conducted by the main researcher, in the outpatient clinics of the Hospital of Clinics of the Federal University of Paraná, linked to SUS (Sistema Único de Saúde – Unified Health System), at a time that met the availability of patients.

For the diagnosis of obesity, the research participants were evaluated according to the growth charts of the World Health Organization (WHO) [19] and the parameters used by the Ministry of Health [18].

Children and adolescents who participated in this study were already part of the care program at the childhood obesity outpatient clinic in order to help with weight loss, but without receiving dietary intervention before the SDSC assessment and the dietary assessment. They only received from the outpatient care team some guidelines on eating habits for the proper monitoring of changes and verification of the results in new appointments.

In order to evaluate the nutrient intake of the patients studied, a previous interview was carried out in order to compare with the information reported in the interview regarding the foods consumed and quantities and with the information reported by the patients in the food record.

To collect energy and macronutrient intake the patients completed the 6-day Food Consumption Record, in which they should record the portions of all foods and beverages consumed, in home measures, (unit, tablespoon, cup, skimmer, ladle, flat plate etc.). After the 6-day Food Consumption Register was returned, the research participants were contacted by telephone to confirm and adjust the data entered so that there would be no doubts about the food consumed and the proper portion. All data collected were transformed into grams and milliliters using the criteria established by the Manual of Food Survey Critiques [20] and then entered into the Erica Program [21] to calculate the macronutrient intake for each meal. The averages of the total calorie intake from six days of consumption for each patient in the sample were used. For statistical analysis, the information found in the collection regarding food intake was compared with reference standards of food consumption in the literature [22].

To investigate the presence of sleep disorders in the population studied we used the Sleep Disturbance Scale for Children (SDSC) questionnaire developed by Bruni et al. (1996) [23], which has been validated for the Brazilian version for children and adolescents aged from 3 to 18 years old [24]. The SDSC is a questionnaire with 26 questions about sleep, covering six months prior to the moment it is applied and presents evaluation scores of 6 aspects inherent to sleep that can lead to the recognition of the risk of the sleep disorders or alterations in children and adolescents. The SDSC questionnaire has good psychometric properties, with a consistency of significance of 0.79 among control cases and 0.71 among clinical cases. Each factor studied has a weight as a score and as a percentage, whose sum can present as a result whether the patient has a risk for sleep disorder or not [[23], [24], [25]]. The SDSC assessment was carried out in an interview with the parents or legal guardians along with the child.

The factors evaluated were Disorders of Initiating and Maintaining Sleep (DIMS), Sleep Breathing Disorders (SBD), Disorders of Arousallnightmares (DA), Sleep Wake Transition Disorders (SWTD), Disorders of Excessive Somnolence (DOES) and Sleep Hyperhidrosis (SHY) [23,24]. The answers coming from the questionnaire were arranged on a Likert scale of five levels, in which the scores with higher values relate to greater severity of sleep symptoms and the T-score>70 was related to the presence of sleep abnormalities in the patients surveyed. This cut-off score was chosen based on Jacquier and Newman [26] in 2018, which can be considered a factor for the presence of sleep disorders.

### 2.2 Statistical analysis

After collecting and recording all data, they were checked and exported to IBM SPSS Statistics version 22 software for statistical analyses.

Categorical variables were expressed as relative (%) and absolute (n) frequencies. All quantitative variables in this study were evaluated using the Shapiro-Wilk normality test and classified according to the mean, standard deviation, median, minimum, and maximum values, depending on the characteristic of the data distribution. The confidence interval considered was 95% (p values < 0.05 were of statistical significance).

Linear Regression was applied to evaluate the association between two or more quantitative variables, and for the association between qualitative, quantitative variables with a binary qualitative outcome, Binary Logistic Regression was applied. For the analysis of the Multiple Regression and Binary Logistic Regression the Correlation Classification according to Finney (1980) [27] was used.

## 3 Results

### 3.1 Demographic and anthropometric characteristics of the participants

The studied group presented a similar number of boys and girls according to Table 1, with a mean age of 10 years and 7 months (±1.95). All participants presented obesity according to the parameters established by WHO for percentile BMI [19].

**Table 1:** Demographic and anthropometric characteristics of children and adolescents with obesity aged 6 to 13 years.

Parameters		Results (N = 43)
Sex	Male	21 (48.8%)
	Female	22 (51.2%)
Age		10.65 (±1.95)
Height/age Z-score		0.77 (±1.54)
BMI (kg/m2)		29.57 (±4.38)
BMI z-*score*		2.85 (1.79–6.26)

The results correspond to absolute (n) and relative (%) frequencies, mean (SD) and median.

### 3.2 Sleep disturbances and the association with food intake habits in children and adolescents

The data collected by applying the SDSC questionnaire showed in Table 2 resulted in the SDSC (total) value of 73 (40–100) points out of a total of 130 points that could be achieved by SDSC when all items obtained a value of 5 on a scale of 1–5. The result found in this study was that 25 children scored for the risk of the presence of the disorder according to the sum of all factors. The other factors evaluated alone demonstrated the risk for the presence of sleep disturbances in the SBD with 22 children at risk for this disorder and in the SWTD with 25 children at risk for the presence of the disorder.

**Table 2:** Data from the SDSC of children and adolescents with obesity aged 6 to 13 years.

Variables	Factor scores (median)	YES N (%)	NO N (%)
SDSC (total)	73 (40–100)	25 (58.1%)	18 (41.9%)
Disorders of Initiating and Maintaining Sleep (DIMS)	60 (41–100)	15 (34.9%)	28 (65.1%)
Sleep Breathing Disorders (SBD)	72 (45–100)	22 (51.2%)	21 (48.8%)
Disorders of Arousallnightmares (DA)	47 (47–100)	9 (20.9%)	34 (79.1%)
Sleep Wake Transition Disorders (SWTD)	75 (41–100)	25 (58.1%)	18 (41.9%)
Disorders of Excessive Somnolence (DOES)	58 (42–100)	17 (39.5%)	26 (60.5%)
Sleep Hyperhidrosis (SHY)	45 (45–93)	11 (25.6%)	32 (74.4%)

The SDSC (total) refers to the median of the sum of the individual scores of the 6 factors. The values in the 3rd and 4th columns correspond to the absolute and relative frequency of the presence of sleep disturbances.

The results in Table 3 correspond to the statistical analysis performed through Binary Logistic Regression to evaluate the association of food intake with sleep disorders. The sum of all SDSC factors showed a positive correlation with the energy intake of 6 days of Food Consumption Record (p = 0.07. r = 0.35) and of macronutrients in percentage values in relation to the total daily intake. As for the SWTD we observed the positive correlation for sex (p = 0.04. r = 0.59) and for the energy intake (p = 0.04. r = 0.59). For the variable Lipids in relation to SWTD, although the results may suggest an association, this was not significant (p = 0.06. r = 0.59). The SBD also showed a positive correlation to sex (p = 0.02. r = 0.43) and to energy intake (p = 0.01. r = 0.43).

**Table 3:** Association between energy intake, sex and macronutrient intake and the risk for the presence of SDSC disturbances among children and adolescents with obesity aged 6 to 13 years.

Independent Variables	SDSC (Total)	SWTD	SBD
Sex	p = 0.02 a∗	p = 0.04 a∗	p = 0.02 a∗
Energy Intake	p = 0.07	p = 0.04 a∗	p = 0.01 a∗
Proteins (%)	p = 0.20	p = 0.17	p = 0.46
Lipids (%)	p = 0.13	p = 0.06	p = 0.28
Carbohydrates (%)	p = 0.17	p = 0.10	p = 0.38
r2 Nagelkerke	r = 0.35	r = 0.59	r = 0.43

SDSC (Total) = Sum of scores for all factors of the Sleep Disturbance Scale for Children; SWTD = Sleep Wake Transition Disorders; SBD = Sleep Breathing Disorders; r = r2 Nagelkerke; ∗ statistical significance for the letter a.

## 4 Discussion

Our findings support the associations of total daily energy intake (TDEI) and the risk of SWTD and SBD. Regarding the influence of diet on sleep, it is important to point out that for the execution of this research it was decided to evaluate the influence of diet on sleep quality and obesity in children and adolescents, since studies evaluating the effect of sleep deprivation and increased consumption leading to obesity are already well established.

In our study the total daily energy intake (TDEI) was positively associated with the risk of SWTD, through the SDSC evaluation, which includes evaluation factors related to both the quality and quantity of sleep. The factors of SDSC evaluated were Disorders of Initiating and Maintaining Sleep (DIMS), Sleep Breathing Disorders (SBD), Disorders of Arousal nightmares (DA), Sleep Wake Transition Disorders (SWTD), Disorders of Excessive Somnolence (DOES) and Sleep Hyperhidrosis (SHY) [23,24].

Ogilvie and Patel [9] suggested that the varying levels of sleep are associated with overweight in children and adults. According to these researchers, individuals who are used to staying awake until late at night, exposed to electronic devices, and who are used to stay more time watching TV, are more prone to low-quality sleep and food intake. In fact, it has been proposed that metabolic adjustments to short-term sleep, such as an increase in Ghrelin blood levels would contribute to obesity [28]. In our study, we proposed to observe the effect of nutrients and energetic intake on sleep quality as it is not of our knowledge that this approach has been explored among children. It is important to mention that, in our study, we did not have a control group to establish a comparison between obese and eutrophic children.

Min et al., in 2018 [7] study found an association between sleep duration and food consumption, with higher energy intake, as well as calorie consumption after 8 p.m. and a tendency to higher calorie consumption in people who sleep less and later. We made an association by means of the Chi-square statistical test considering the time of the patient's last meal (after 9 p.m. and after 10 p.m.) and the presence of sleep disorders according to the SDSC (SBD and SWTD), and we did not find an association between these variables. However, this fact does not rule out the possibility that there is an association between the quality of food before bedtime and the quality of sleep of these patients.

In 2021, Saidi et al. [29], verified changes in sleep patterns in adolescents exposed to a controlled eucaloric diet as compared to a group that maintained their habitual diet (ad libitum). The research showed that adolescents with obesity had worse sleep quality as compared to other adolescents with adequate weight for both eucaloric and ad libitum intake. Although we did not perform the analysis with a control group and only considered their habitual diet (ad libitum), the patients studied in our research were at risk for SWTD and SBD. In the Saidi et al. [29] study it was also observed that excess energy intake had a negative impact on sleep quality the latter the meal was ingested and De Melo et al., in 2019 [30] report in their study the association between higher food intake at night and worse sleep in adult patients with obstructive sleep apnea whose associations were not found in our study.

Other studies carried out with the adult public, the association of food intake with sleep disorders was explored. Wilson et al., in 2022 [31] reviewing observational and interventional studies, suggested that healthier diets improve positively sleep quality. They described studies showing an association between high-carbohydrate diets (with carbohydrate content >50% and <80%) and very high-carbohydrate diets (with carbohydrate content >80%) with worsening sleep quality, especially shortening deep sleep. The proportion of simple and complex carbohydrate intake was also associated with sleep quality, with higher fiber intake reflecting better sleep quality and higher sugar consumption leading to a worsening in sleep quality. Higher consumption of saturated fat had also a negative effect on sleep and the consumption of polyunsaturated fats allowed for an improvement in sleep patterns. Regarding our study, the association we found was between total energy consumption and SWTD and SBD, and not with the macronutrients.

St-Onge et al., in 2016 [32] reported the association between fiber and saturated fat intake throughout the day with worsening sleep patterns, with the characteristic of lighter and less deep sleep. They also found an association between the proportion of energy consumed throughout the day from sugar and non-sugar or fiber types of carbohydrates and a greater number of nighttime awakenings, however, these variables were not evaluated in our study.

In another analysis of this study, 58.1% (n = 25) of the sample presented risk for sleep disturbance demonstrated by the sum of the scores of all the SDSC sleep characteristics analysis factors with a median of 73 (40–100). This value of 70% called pathological sleep was validated by Bruni et al., in 1996 [23]. Jacquier and Newman [26] in 2018, updated this parameter with contemporary data enabling the use for current studies. Considering that, in our study, all involved were with obesity, this result could suggest that the risk for sleep disorders would be related to the diet characteristics of this sample.

According to El and Nunes [28] the duration of sleep is associated not only to body weight gain, but also to the feeding profile with changes in appetite, possibly being influenced by the increased levels of the ghrelin hormone that stimulates hunger and reduction of the leptin hormone that brings the feeling of satiety. This change occurred mainly in the age group of preschool children. In our study it was not possible to evaluate hormonal factors in the patients, but it should be considered that hormonal changes from reduced number of hours of sleep, for example, could affect food intake and result in obesity. Thus, poor eating habits and the resulting excess weight could lead to metabolic changes that alter the quantity and quality of sleep.

Another factor to be considered is the propensity to consume more caloric food when we are sleep deprived, in this sense, Ward and collaborators [6], reinforce that the lack of sleep can lead to greater consumption of lower quality food, that is, more processed and with a higher glycemic index. We did not analyze the glycemic index of the carbohydrate ingested, especially because de glycemic index of diverse food ingested by our study population is lacking in the literature. Although it makes it difficult to identify specific food that would impact glycemic control, we can refer that the patients in our study tend to have a diet in the normal range of carbohydrate intake recommendations and eucaloric diets.

St-Onge [10] sought to associate higher calorie intake with sleep disorders and observed that this was associated with greater daytime sleepiness, while sleep disorders, such as insomnia, obstructive sleep apnea or a combination of the two, were related to lower carbohydrate intake when compared to those who did not have the disorders or without obesity, different from our study where patients had the presence of SBD but with lower than recommended energy intake and carbohydrate intake was adequate according to the literature (45–65%/TDEI) and in our study the value of the average intake of carbohydrates showed the result of 45.42% of total daily energy intake [22].

In the study by Goldschmidt et al. [8] which had as its main objective to evaluate the duration and quality of sleep related to eating behavior in children and youth, positive associations were found regarding energy intake in relation to total sleep time. Although the study evaluated established an analysis parameter that we did not use in this study, which was the evaluation of the overall quality of the diet, it is interesting to note the conclusion they reached when they demonstrated that each additional hour of sleep was associated with lower consumption of calories from solid fats, alcohol, and sugar.

In addition to the diverse cycle of life, we should point out that, the diverse manner to analyze the variable sleep such as quality of sleep, deep of sleep, sleep duration, and nighttime awakenings, decreases the feasibility of comparing our result to other publications.

Due to the need to find other means to control the progression of obesity in children and adolescents, we consider that further studies should be carried out with larger samples and with the use of other objective and subjective methods of sleep assessment and food consumption in order to better explore the association of food consumption and sleep, seeking alternatives to correct inadequate eating habits that interfere with sleep quality.

## 5 Conclusion

In conclusion to the analysis of the study presented, it was possible to find the association between food intake and SBD and SWTD through the analysis of the SDSC sleep characteristics, in which the positive association between food intake and the risk for sleep disorders was demonstrated.

The positive association of food intake with SWTD and SBD may suggest that there is an influence the dietary habits on the development of sleep disorders in the population studied.

## Funding

This research did not receive any specific grant from funding agencies in the public, commercial, or not-for-profit sectors.
