# Erratum to “Association of food intake with sleep disorders in children and adolescents with obesity” [Sleep Med: X 4 (2022) 100053]

**DOI:** 10.1016/j.sleepx.2024.100132

**Published:** 2024-11-16

**Authors:** Raquel S.M. Zarpellon, Dra Regina M. Vilela, Fernando Mazzilli Louzada, Dra Rosana B. Radominski, Dra Ana Chrystina de Souza Crippa

**Affiliations:** a Postgraduate Program in Child and Adolescent Health, Hospital de Clínicas, Federal University of Paraná, Curitiba, Paraná, Brazil; b Federal University of Paraná (UFPR), Health Sciences Sector, Department of Nutrition, Curitiba, Paraná, Brazil; c Federal University of Paraná (UFPR), Department of Physiology, Curitiba, Paraná, Brazil; d Federal University of Paraná (UFPR), Health Sciences Sector, Curitiba, Paraná, Brazil; e Neuropediatrics Center (CENEP), Hospital de Clínicas, Federal University of Paraná, Curitiba, Paraná, Brazil

The publisher regrets “Declaration of conflict of interest” was not included in the published article that appeared in the previous issue of Sleep Medicine: X.

## Declaration of competing interest

The authors declare that they have no known competing financial interests or personal relationships that could have appeared to influence the work reported in this paper.

The publisher would like to apologise for any inconvenience caused.

